# Real time analysis of β_2_-adrenoceptor-mediated signaling kinetics in Human Primary Airway Smooth Muscle Cells reveals both ligand and dose dependent differences

**DOI:** 10.1186/1465-9921-12-89

**Published:** 2011-07-02

**Authors:** Charlotte K Billington, Ian P Hall

**Affiliations:** 1 Division of Therapeutics and Molecular Medicine, Nottingham Respiratory Biomedical Research Unit, Floor D, South Block, University Hospital of Nottingham, The University of Nottingham, Nottingham, NG7 2UH, UK

## Abstract

**Background:**

β_2_-adrenoceptor agonists elicit bronchodilator responses by binding to β_2_-adrenoceptors on airway smooth muscle (ASM). *In vivo*, the time between drug administration and clinically relevant bronchodilation varies significantly depending on the agonist used. Our aim was to utilise a fluorescent cyclic AMP reporter probe to study the temporal profile of β_2_-adrenoceptor-mediated signaling induced by isoproterenol and a range of clinically relevant agonists in human primary ASM (hASM) cells by using a modified Epac protein fused to CFP and a variant of YFP.

**Methods:**

Cells were imaged in real time using a spinning disk confocal system which allowed rapid and direct quantification of emission ratio imaging following direct addition of β_2_-adrenoceptor agonists (isoproterenol, salbutamol, salmeterol, indacaterol and formoterol) into the extracellular buffer. For pharmacological comparison a radiolabeling assay for whole cell cyclic AMP formation was used.

**Results:**

Temporal analysis revealed that in hASM cells the β_2_-adrenoceptor agonists studied did not vary significantly in the onset of initiation. However, once a response was initiated, significant differences were observed in the rate of this response with indacaterol and isoproterenol inducing a significantly faster response than salmeterol. Contrary to expectation, *reducing *the concentration of isoproterenol resulted in a significantly *faster *initiation of response.

**Conclusions:**

We conclude that confocal imaging of the Epac-based probe is a powerful tool to explore β_2_-adrenoceptor signaling in primary cells. The ability to analyse the kinetics of clinically used β_2_-adrenoceptor agonists in real time and at a single cell level gives an insight into their possible kinetics once they have reached ASM cells *in vivo*.

## Introduction

Frontline drugs in the treatment of asthma and chronic obstructive pulmonary disease (COPD) target β_2_-adrenoceptors on airway smooth muscle cells and thereby elicit a bronchodilatory response. These drugs are grouped into short-acting and long-acting β_2_-adrenoceptor agonists (SABAs and LABAs respectively) [1]. SABAs include salbutamol (albuterol) whilst LABAs include salmeterol and terbutaline. Formoterol can be considered as both short-acting and long-acting due to its rapid onset and long duration of action [2]. Recently a new category was created to encompass the Ultra-long-acting β_2_-adrenoceptor agonists such as indacaterol [3].

Following binding of agonist to β_2_-adrenoceptors on airway smooth muscle, a well-documented signaling cascade is initiated resulting in activation of adenylyl cyclase, cyclic AMP formation, protein kinase A (PKA) activation and ultimately airway relaxation [4]. In addition to PKA activation, cyclic AMP also binds to and activates Epac whose functional role in airway smooth muscle cells has recently received significant scrutiny with investigators attempting to dissect PKA- versus Epac-mediated functional outputs [5-9].

*In vivo*, the time between drug administration and bronchodilation varies significantly depending on the β_2_-adrenoceptor agonist used (e.g. < 2 minutes for salbutamol compared to ~30 minutes for salmeterol) [2]. Much of this variability, particularly with respect to salmeterol, is hypothesised to be due to pharmacokinetic properties governing distribution in the airways where more lipophilic compounds become retarded by successive cell membranes (Sears *et al.*, 2005). Whilst it is easy to measure the total time between drug administration and clinical effect *in vivo *for a range of β_2_-adrenoceptor agonists, it is much more difficult to decipher the mechanisms responsible for the observed differences between agonists.

In this study we aimed to quantify agonist-specific differences in the onset of β_2_-adrenoceptor activation at the membrane of the target cell, the human airway smooth muscle cell. Our aim was also to investigate in real time the lag between the addition of β_2_-agonist to the buffer surrounding the cell and the initiation of β_2_-adrenoceptor-mediated signaling in addition to the subsequent rate of this response. For the studies described in this paper, we chose to use a previously reported and well-characterised fluorescent reporter probe as a readout for β_2_-adrenoceptor-mediated signaling designed, cloned and characterised by Jalink and colleagues, namely CFP-Epac(dDEP, CD)-VENUS [10,11]. This probe is homogenously distributed throughout the cell thus cyclic AMP responses can be imaged in a global manner. The probe was visualised in real time on a spinning disk confocal microscope which, in contrast to laser scanning confocal microscopes, does not require extensive corrective measurements and algorithms to overcome the instability of excitation sources and counteract the manipulation by the user of pinhole size, detector gain, amplifier offset, amplifier gain and excitation intensities between experiments. In addition, as the probe described is a single polypeptide and thus has fixed stoichiometry of fluorescent proteins, we were able to quantify emission ratioing as can be done with a widefield microscope, however with the advantage of acquiring high quality confocal images (discussed in [12]. The resulting timecourse reflecting the conformational change of the probe due to the binding of cyclic AMP was analysed in terms of (1) time lag between the direct addition of drug into the buffer surrounding the cell and the initiation of a measurable response by the probe (i.e. an increase in the emission ratio from baseline) and (2) the rate of this response. Analyses involved comparing responses to isoproterenol and a range of clinically relevant β_2_-adrenoceptor agonists.

Here we report that when the initiation of Epac reporter activity was assessed, there was no difference between the β_2_-adrenoceptor agonists studied. However, in terms of the rate of response, indacaterol and isoproterenol were observed to be faster than salmeterol whereas salbutamol and formoterol exhibited intermediate rate of response times. When the kinetics of different concentrations of isoproterenol were compared surprisingly the time between direct drug addition and an initiation of a response was significantly slower with higher concentrations of isoproterenol.

## Methods

### Human Airway Smooth Muscle Cell culture

Human airway smooth muscle (hASM) cells were prepared as previously described either from explants of trachealis muscle obtained from individuals free of respiratory disease [13] or via enzymatic dispersion from individuals undergoing thoracotomy [14]. Ethical approval was obtained from the Nottingham Local Ethical Research Committee (EC00/165). Cells were routinely cultured in Dulbecco's modified Eagles media (DMEM) containing 10% fetal calf serum and glutamine (2 mM) and incubated at 37°C in 5% CO_2 _and 95% air. Cells isolated and cultured in this way have been extensively characterised and shown to retain many of the phenotypic properties of freshly isolated airway smooth muscle cells [15,16].

### Transfection of hASM cells

hASM cells were allowed to reach 50-80% confluency prior to transfection and then incubated for a further 24-48 h. Lipofectamine 2000 (Invitrogen, Paisley, UK) was used as the transfection reagent as per manufacturer's instructions. Three microlitres of Lipofectamine 2000 and 1 μg CFP-Epac(dDEP,CD)-VENUS were used per 35 mm diameter glass-bottomed dish.

### Imaging Fluorescence Resonance Energy Transfer (FRET)

Primary hASM cells were seeded into 35 mm No.0 glass-bottomed dishes (Mattek Corp., Ashland, MA, USA) and transfected as described above. Cells selected for imaging had similar levels of fluorescence and displayed a diffuse distribution of CFP-Epac(dDEP,CD)-VENUS throughout the cell. For imaging purposes cells were rinsed twice with KREBs buffer (118 mM NaCl, 4.7 mM KCl, 1.2 mM MgSO_4_, 1.3 mM CaCl_2_, 1.2 mM KH_2_PO_4_, 4.2 mM NaHCO_3_, 10 mM HEPEs, 11.7 mM Glucose) prior to 2 ml KREBs buffer being added to the well. As required drugs were added to the well in 20 μl volumes. Cells were imaged on an inverted microscope (Zeiss AxioObserver) with widefield and spinning disk confocal capabilities (imaging system including Yokogawa CSU22 spinning disk confocal head assembled by Improvision, Perkin Elmer, Coventry, UK). All studies utilised a 63× water immersion objective (1.3 numerical aperture, Zeiss). For confocal images, cells were excited with a laser emitting at 440 nm and the emission of CFP and YFP were detected by rapid switching of 470 nm and 535 nm bandpass filters positioned in a filter wheel and the FRET ratios measured as changes in the 470 nm/535 nm emission intensities. Images were acquired and processed with Volocity 5.0 software (Improvision, Perkin Elmer, Coventry, UK).

### Cyclic AMP Assay

Tritiated cyclic AMP production was assayed using a prelabeling assay exactly as previously described [13].

### Data Analysis and Statistical Procedures

Differences between the results following exposure to β_2_-adrenoceptor agonists were compared by analysis of variance (ANOVA) in conjunction with Bonferroni's post hoc test. Figures represent mean values (± SEM). Statistical analyses, curve fitting and generation of EC_50 _data were performed by using GraphPad Prism v5 (GraphPad, San Diego, CA, USA); a P-value < 0.05 was considered significant.

### Materials

All chemicals were analytical grade or higher. Plasticware was from Costar (High Wycombe, UK). Glass-bottomed dishes were from Mattek (Ashland, MA, USA). All chemicals and reagents were purchased from Sigma-Aldrich (Poole, UK) unless otherwise stated. The CFP-Epac(dDEP,CD)-VENUS construct was a kind gift from Dr Kees Jalink (NKI, Amsterdam, Netherlands). Lipofectamine 2000 was from Invitrogen (Paisley, UK).

## Results

### Real time emission ratio imaging of Epac-based probe

In a previous publication, we described how β_2_-adrenoceptor-mediated cyclic AMP signaling was assessed in hASM cells by visualising and measuring changes in ratio imaging between CFP and YFP variant fluorophores fused to Epac1 [17]. We were able to extend these studies by utilising a higher magnification lens, a more sensitive camera and by transferring the studies to a confocal rather than epifluorescent microscope. Whilst it is possible to quantify Fluorescence Resonance Energy Transfer (FRET)/emission ratioing using a classic laser point scanning microscope, this is associated with a number of challenging technical issues (see introduction). In these studies we therefore used a spinning disk confocal microscope (see methods) which allows alterations in the emission ratio to be assessed at high magnification and high speed. It is particularly suited to use with single-polypeptide FRET sensors and allows changes in emission ratio to be directly quantified and traces to be visualised live.

**Figure 1:**
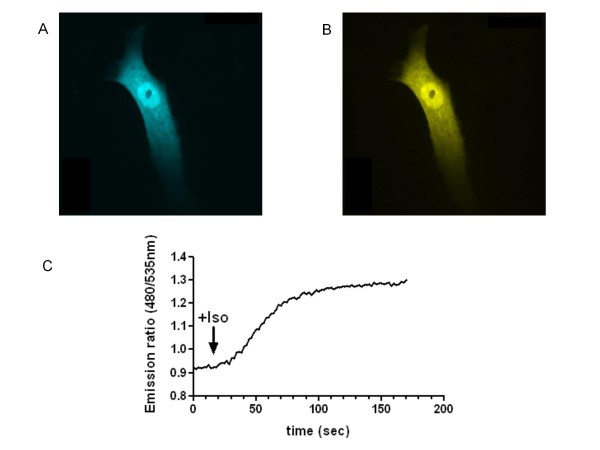
**Isoproterenol-induced changes in cyclic AMP activity in a single hASM cell imaged via confocal microscopy using the altered emission profile of CFP-Epac(dDEP,CD)-VENUS as a readout**. CFP-Epac(dDEP,CD)-VENUS expression 48 hours post-transfection is shown in panel A (CFP excited with 440 nm and 480 nm CFP emission fluorescence recorded). Panel B shows uncorrected FRET in the same cell under basal conditions (CFP excited with 440 nm and 535 nm FRET emission fluorescence recorded). Panel C is a representative trace of the altered emission ratio (essentially a change in FRET) in real time in response to 10 μM isoproterenol (+ Iso).

The single polypeptide cytosolic probe utilised in these studies termed CFP-Epac(dDEP,CD)-VENUS, was produced by the Jalink group and has been described previously [10,17]. Figure 1A shows CFP emission following excitation at 430 nm in a typical hASM cell 48 hours after transfection with CFP-Epac(dDEP,CD)-VENUS and confirms the cytosolic distribution of the probe as reported by ourselves and others [10,17]. As under basal conditions the CFP and YFP-variant (Venus) fluorophores of CFP-Epac(dDEP,CD)-VENUS are in close proximity FRET occurs. Figure 1B shows an uncorrected image of this i.e. YFP emission following excitation of CFP at 430 nm in the same hASM cell under basal conditions.

Exposure to elevators of cyclic AMP, in this case the non-selective β-adrenoceptor agonist, isoproterenol (10 μM), results in the binding of cyclic AMP to Epac and a subsequent conformational change of Epac leading to an increased distance between the CFP and YFP termini of the probe and a subsequent loss of FRET. The trace of the changes in emission ratio are shown in Figure 1C. Traces such as this were used to extract the data shown in the majority of the figures in this manuscript. As hASM cells solely express the β_2_-adrenoceptor subtype, all responses to isoproterenol essentially reflect β_2_-adrenoceptor activation [16].

### Pharmacological profile of isoproterenol determined via confocal FRET

**Figure 2:**
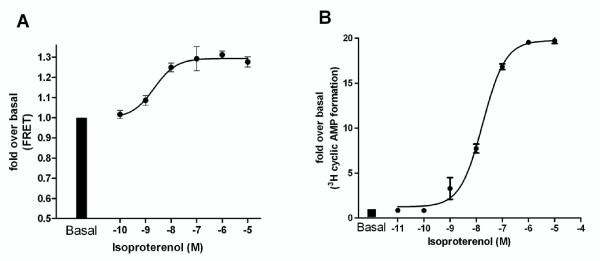
**Cyclic AMP activation induced by a range of doses of isoproterenol in hASM cells using (A) CFP-Epac(dDEP,CD)-VENUS activation or (B) ^3^H-cyclic AMP formation as a readout**. (A) For Epac-based studies single hASM cells expressing CFP-Epac(dDEP,CD)-VENUS were excited at 440 nm and the emission ratio (470/535) changes collected in real time. The maximal ratio was recorded and plotted. Data are expressed as fold over basal. Each data point represents the mean (± SEM) of 3-9 separate experiments. (B) For studies investigating ^3^H-cyclic AMP formation, monolayers of hASM cells in 24 well plates were labelled with ^3^H-adenine for 2 hours and then exposed to the appropriate concentrations of isoproterenol for 5 minutes. The reaction was terminated by addition of hydrochloric acid and total ^3^H-labelled cyclic AMP was collected via column-based separation and quantified by scintillation counting [13]. Data are expressed as fold over basal. Each data point represents the mean (± SEM) of 3-6 experiments.

Previous studies using widefield imaging of the Epac-based probe had enabled us to produce concentration response curves to isoproterenol by perfusing a single cell with increasing doses of drug, allowing time for cellular recovery after each dose [17]. We extended these studies to see whether a full concentration curve could be produced from confocal FRET data. As confocal imaging only utilises fluorescent signals from a thin optical slice rather than the entire thickness of the cell (as in widefield imaging) we were concerned that the vastly reduced number of fluorescent events that would be measured confocally may give variable data. Despite the technically challenging nature of these experiments and the potential for inter-cell variability, it was possible to produce a concentration response curve to isoproterenol as shown in Figure 2A (pEC_50 _= 8.6 ± 0.25, n = 3-9). When compared with a concentration response curve produced by a traditional radiolabeling assay (Figure 2B), it is apparent that maximal Epac probe activation occurs at concentrations of isoproterenol which are submaximal in terms of whole cell cyclic AMP measured using radiolabeling assays (pEC_50 _= 7.75 ± 0.07, n = 3). This implies probe saturation may be occurring at concentrations of isoproterenol above around 10^-7 ^M and 10^-6^M.

### Pharmacological profile of a range of β_2_-adrenoceptor agonists determined via confocal emission ratio imaging

**Figure 3:**
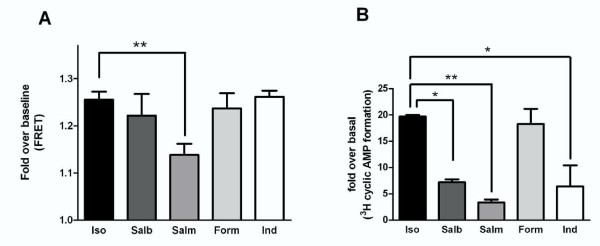
**Cyclic AMP activation induced by single doses of a range of β_2_-adrenoceptor agonists in hASM cells using (A) CFP-Epac(dDEP,CD)-VENUS activation or (B) ^3^H-cyclic AMP formation as a readout**. β_2_-adrenoceptor agonists studied were isoproterenol (10 μM), salbutamol (1 μM), salmeterol (100 nM), indacaterol (1 μM) and formoterol (1 μM). (A) For FRET-based studies single hASM cells expressing CFP-Epac(dDEP,CD)-VENUS were excited at 440 nm and the emission ratio (470/535) changes collected in real time. The maximum ratio observed was recorded and plotted. Data are expressed as fold over basal. Each data point represents the mean (± SEM) of 6-16 separate experiments. **denotes P < 0.01. (B) For studies investigating ^3^H-cyclic AMP formation, monolayers of hASM cells in 24 well plates were labelled with ^3^H-adenine for 2 hours and then exposed to the appropriate concentrations of β_2_-adrenoceptor agonist for 5 minutes. The reaction was terminated by addition of hydrochloric acid and total ^3^H-labelled cyclic AMP was collected via column-based separation and quantified by scintillation counting [13]. Data are expressed as fold over basal. Each data point represents the mean (± SEM) of 3-9 experiments. * denotes P < 0.05, **denotes P < 0.01.

In addition to isoproterenol, we assessed the fold increase in confocal emission ratio imaging in response to a range of clinically relevant β_2_-adrenoceptor agonists at concentrations we have previously shown to be maximal for cyclic AMP formation [18] namely salbutamol (1 μM), salmeterol (100 nM), formoterol (1 μM) and indacaterol (1 μM) (Figure 3). When confocal emission ratioing was utilised as a readout, only salmeterol produced a significantly smaller response than isoproterenol (10 μM) suggesting, as highlighted in the previous section, that the Epac probe is maximally activated at lower concentrations than are maximal for whole cell cyclic AMP formation.

### Temporal profile of a range of β_2_-adrenoceptor agonists

**Figure 4:**
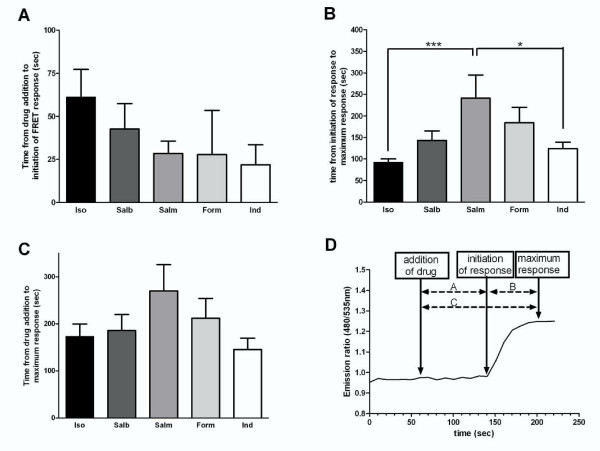
**Time taken for a range of β_2_-adrenoceptor agonists to (A) initiate an increase in CFP-Epac(dDEP,CD)-VENUS activation and (B) to induce a maximal response c.f. initiation**. The time between response initiation and maximal response is shown in C. The labelled trace in D depicts how these data were obtained from real time traces. The β_2_-adrenoceptor agonists studied were isoproterenol (10 μM), salbutamol (1 μM), salmeterol (100 nM), indacaterol (1 μM) and formoterol (1 μM). Single hASM cells expressing CFP-Epac(dDEP,CD)-VENUS were excited at 440 nm and the emission ratio (470/535) changes collected in real time. Each data point represents the mean (± SEM) of 6-19 experiments. * denotes P < 0.05, ***denotes P < 0.001.

One major advantage of the described Epac-based studies utilising the spinning disk imaging system is the ability to measure β_2_-adrenoceptor-mediated responses at high speed and in real time. Thus given the clinical differences in rate of onset of action of these drugs we explored the rate of cyclic AMP-induced Epac activation that the different β_2_-adrenoceptor agonists could achieve. Previous attempts to look at early time points using whole cell cyclic AMP assays have been problematic due to the technical constraints of the assay. Rapid imaging (5 second intervals) using the Epac probe allows this problem to be addressed. Temporal analyses included measuring (1) time lag between the direct addition of drug into the buffer surrounding the cell and the initiation of a measurable response by the probe (Figure 4A), (2) time between the initiation of the response and the time at which this was maximal (Figure 4B) and (3) a combination of (1) and (2), i.e. the total time between drug addition and maximal effect (Figure 4C). Figure 4D depicts which part of the response was analysed for each set of results.

No significant difference was observed between β_2_-adrenoceptor agonists in the time between direct addition of the drug and the initiation of a response. Interestingly however, salmeterol, despite its known slow duration of onset of bronchodilation, exhibited a trend towards a faster onset of response than most of the β_2_-adrenoceptor agonists studied although this did not reach significance. Significant differences were observed in the times taken for a response to reach a maximal point with salmeterol being significantly slower than isoproterenol and indacaterol whilst salbutamol and formoterol were intermediate to these (Figure 4B). When the total time between drug addition and maximal response was assessed, no significant difference was observed between β_2_-adrenoceptor agonists, and all agents achieved maximum activation within 5 minutes.

### Analysis of the rate of β_2_-adrenoceptor-agonist mediated responses

**Figure 5:**
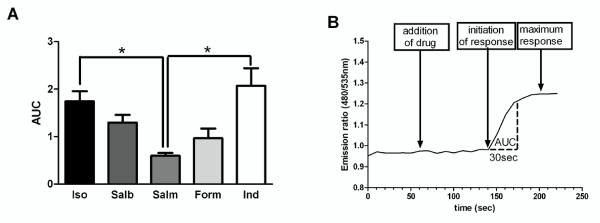
**Re-analysis of data shown in figure 4 concentrating on the response observed in the first 30 seconds after initiation i.e. at a timepoint whereby probe saturation has not occurred**. Data was analysed via (A) Area Under the Curve (AUC). B outlines the region utilised for these additional analyses. Each data point represents the mean (± SEM) of 6-19 experiments.* denotes P < 0.05, ** denotes P < 0.01.

To ensure that the differences observed in Figure 4 could not solely be explained by saturation of the Epac probe, the first 30 seconds of each response was analysed. It was observed that in this time period none of the β_2_-adrenoceptor agonists studied had achieved maximal activation and hence we were satisfied that the probe was not saturating in this time period. This initial response was analysed both by area under the curve (AUC) (Figure 5A) and ratio change per minute. The trends observed in Figure 4 were still apparent following these analyses with salmeterol causing β_2_-adrenoceptor-induced probe activation at a significantly slower rate than isoproterenol and indacaterol and with the response to salbutamol being intermediate to these. When ratio change per minute was analysed again salmeterol exhibited a significantly slower change than isoproterenol and indacaterol (n = 6-13, p < 0.01 for both comparisons). In addition, formoterol demonstrated a significantly slower increase in probe activation compared to isoproterenol in terms of ratio change per minute (n = 6-13, p < 0.05).

### *Lower *concentrations of isoproterenol initiate Epac-based probe activation *faster *than higher concentrations

**Figure 6:**
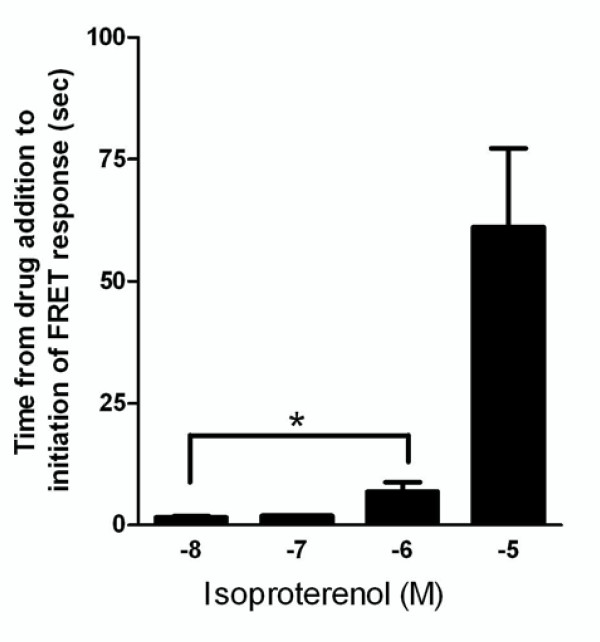
**Time taken for a range of concentrations of the β_2_-adrenoceptor agonist isoproterenol (10^-8^M-10^-5^M) to initiate an increase in CFP-Epac(dDEP,CD)-VENUS activation**. These data were obtained from real time traces comparable to those depicted in Figure 4D. Single hASM cells expressing CFP-Epac(dDEP,CD)-VENUS were excited at 440 nm and the emission ratio (470/535) changes collected in real time. Each data point represents the mean (± SEM) of 4-19 experiments. * denotes P < 0.05.

Having studied the temporal profile of different β_2_-adrenoceptor agonists, finally we investigated whether agonist concentration impacted response times (Figure 6). Contrary to expectation it was determined that higher concentrations of isoproterenol were significantly slower to initiate a β_2_-adrenoceptor-mediated response. However, this difference was observed in the time lag between the point of drug addition and the response initiation as oppose to between drug initiation and maximal response. The difference in response was large, with 10^-5 ^M isoproterenol taking almost 10 times as long to initiate a response compared with 10^-6 ^M (p < 0.05, n = 6-19). This does not appear to be a non-specific effect seen only at high doses as a further concentration-dependent decrease in the rate of onset of activation was observed between hASM cells exposed to10^-6 ^M versus 10^-8 ^M isoproterenol (p < 0.05, n = 6).

## Discussion

In this paper we describe β_2_-adrenoceptor-mediated activation of the Epac-based probe, CFP-Epac(dDEP,CD)-VENUS in human primary airway smooth muscle (hASM) cells. By transfecting this probe into hASM cells, we were able to image and quantify pharmacological and temporal information regarding cyclic AMP activity following exposure to a range of β_2_-adrenoceptor agonists. We chose to utilise this monomeric Epac-based probe in preference to the multimeric PKA-based probe initially utilised by Zaccolo and colleagues [19] mainly as only monomeric reporter probes are suitable for the high speed emission ratio imaging experiments required for this study [12] but also as the Epac-based probe has been reported to have a larger dynamic range and to be sensitive to lower concentrations of cyclic AMP than the PKA-based probe [11]. As highlighted in the introduction, the spinning disk confocal imaging system offers major advantages over the commonly used laser scanning confocal microscopes when used for real time visualisation and quantification of FRET-based probes in live cells and although it produces confocal images, in terms of simplicity of data analysis and controls, it can be considered as being equivalent to widefield systems. It is thus not subject to the technical issues affecting laser scanning confocal microscopes which have been described previously [12,20]. The spinning disk system is also particularly powerful in terms of the ability to assess Epac activation at short time intervals (time interval between image acquisition is ~5 seconds in the studies shown in this paper) which has allowed the dynamics of the onset of these agents to be assessed accurately in this hASM cultured cell system.

The concept of different ligands inducing diverse responses via the same G-protein coupled receptor (GPCR) in terms of magnitude of effect (i.e. full *versus *partial agonists) has been accepted for many years. It is now increasingly clear, however, that there are also ligand-dependent differences in the extent to which disparate signaling pathways are activated despite the same GPCR being activated. Perhaps the most extreme example of this was seen in a study by Woo *et al.*, who observed different isomers of fenoterol to drive endogenous β_2_-adrenoceptors to signal via Gs- or Gi-proteins to a different extent. Specifically S,R isomers of fenoterol acting at β_2_-adrenoceptors in adult rat cardiomyocytes were observed to signal to a greater extent via Gi-mediated ERK activation when compared with R,R isomers whilst the converse was observed when Gs-mediated effects were quantified [21]. As this phenomenon has become more widely reported it has attracted a variety of labels including biased agonism, conformational selection, stimulus trafficking, ligand-directed signaling and ligand functional selectivity; however a consensus term has not yet been chosen [22]. It has also been the subject of a number of reviews [23-27] with ligand-directed signaling specifically at β-adrenoceptors being systematically reviewed by Evans *et al.*, last year [22]. As outlined in these reviews, the accepted explanation for ligand-directed signaling is the ability of diverse ligands to induce varied conformational receptor states resulting in the favouring of one downstream pathway over another which has intriguing therapeutic implications.

Despite the recent expansion of studies investigating ligand-directed signaling in terms of response size, downstream effectors, and subsequent receptor desensitiation or internalisation, there are limited reports of the kinetics of these effects and only one utilise endogenous β_2_-adrenoceptors in human primary cells [28]. In their review, Evans *et al.*, [22] highlighted the importance of confirming "proof of principle" studies of ligand-directed signaling in recombinant systems by utilising more therapeutically relevant models where receptors are present at nearer physiological levels and we believe that the single cell imaging of hASM cells described in this manuscript provided an ideal system in which to investigate ligand-directed signaling at endogenous β_2_-adrenoceptors.

Utilising HEK293-GloSensor cells and human bronchial smooth muscle cells both endogenously expressing β_2_-adrenoceptors Rosethorne and colleagues reported a correlation between intrinsic agonist efficacy and rate of cyclic AMP generation [28]. Lohse and colleagues also observed partial agonists to elicit slower responses than full agonists in their studies into the kinetics of recombinant β_2_-adrenoceptor activation in HEK293 and CHO cells following exposure to both endogenous (epinephrine and norepinephrine) and synthetic (isoproterenol, fenoterol and terbutaline) ligands [29]. This finding echoed previous work from the same group when the α_2A_-adrenoceptor was investigated [30].

In our studies, significant ligand-dependent differences in the kinetics of the signaling response of endogenous β_2_-adrenoceptors were also observed. In terms of the time lag between drug addition and initiation of response, higher efficacy did not appear to translate into faster response initiation, indeed it was interesting to note that the partial agonist salmeterol initiated a response as rapidly as the other agonists studied (Figure 4A). However, salmeterol was significantly slower to reach a maximal response implying that this phase of signaling could be dependent upon drug efficacy. This was perhaps surprising as it occurred despite salmeterol requiring a smaller rise in ratio change compared to isoproterenol before a maximum response was observed i.e. at maximal activity salmeterol induced a 1.13 fold increase over baseline whereas maximal probe activation was induced by isoproterenol 1.25 fold over baseline (see Figure 3).

In addition to the correlation between efficacy and rate of cyclic AMP response, Rosethorne et al discussed the link between rate of response and the lipophilicity of each compound with the expectation being that increased lipophilicity would correlate with a slower rate of response. Although salmeterol and indacaterol exhibit almost identical lipophilicity, salmeterol demonstrated a slower onset of action than indacaterol and indacaterol induced cyclic AMP formation at an even faster rate than the hydrophilic salbutamol which led the authors to conclude that efficacy was a stronger determinant of clinical onset than lipophilicity.

Our data also does not support increased lipophilicity of the agonist as being the key driver for a slower rate of onset and, as mentioned above, salmeterol was observed to initiate a response as rapidly as all the other agonists studied (Figure 4A). Interestingly there is a trend towards the more lipophilic drugs (indacaterol, salmeterol) initiating a rate of response faster than the hydrophilic ones (isoproterenol, salbutamol).

One very surprising finding from our data was the concentration-dependent effect of isoproterenol on the rate of β_2_-adrenoceptor-mediated Epac probe activation. Two very recent studies report temporal data of β_2_-adrenoceptor-mediated activity and each of these showed an increased rate of response correlating with increased agonist concentration [29,31]. The *initiation *of response in both of these studies appeared to be concentration-independent although this may be an incorrect conclusion to draw as the traces may be normalised such that the initiation of response is at the same point to allow comparison of response magnitude. Schroder *et al.*, utilised a novel technique based on dynamic mass redistribution (DMR) of cellular constituents to quantify GPCR signaling in real time in live cells and representative data shown from 4 experiments in CHO cells overexpressing β_2_-adrenoceptors appears to show a quicker response to agonist (orciprenaline) at higher concentrations than lower ones although any statistical analysis of this was not commented on.

A possible explanation for the slower response at high concentrations of agonist is that an increased proportion of β_2_-adrenoceptors with bound ligand essentially overwhelm the downstream cascade inducing a negative feedback mechanism which slows down the rate at which the pathway is activated without altering the extent to which it is activated. This negative feedback mechanism could be GRK- or arrestin- or phosphodiesterase-mediated or could potentially be due to a split in the cyclic AMP-driven signal between PKA and Epac. Determining whether the observed phenomenon is observed with clinically relevant β_2_-adrenoceptor agonists and elucidating the mechanisms involved requires additional investigation.

In summary therefore we describe for the first time a confocal based imaging assessment of the real time kinetics of β_2_-adrenoceptor-mediated Epac activation in primary cultured hASM cells. Both ligand- and concentration-dependent differences in kinetics were observed and these data suggest that the delayed onset of bronchodilation seen with salmeterol is not related to altered pharmacodynamic effects at the cell surface, but is probably due to pharmacokinetic effects governing distribution in the airways as has previously been suggested [2].

These approaches provide valuable insights into the mechanism of activation of downstream pathways stimulated by β_2_-adrenoceptor agonists, and are potentially extendable into other primary cell systems.

## Abbreviations

hASM: human airway smooth muscle; cAMP: cyclic AMP; Epac: Exchange Protein directly Activated by Cyclic AMP; CFP: Cyan Fluorescent Protein; YFP: Yellow Fluorescent Protein; FRET: Fluorescence Resonance Energy Transfer.

## Competing interests

The authors declare that they have no competing interests.

## Authors' contributions

CKB participated in research design, conducted experiments, performed data analysis and drafted the manuscript. IPH conceived of the study, participated in research design and revised the manuscript critically. Both authors read and approved the final manuscript.
